# Layer-specific anatomical and physiological features of the retina’s neurovascular unit

**DOI:** 10.1016/j.cub.2024.11.023

**Published:** 2024-12-16

**Authors:** William N. Grimes, David M. Berson, Adit Sabnis, Mrinalini Hoon, Raunak Sinha, Hua Tian, Jeffrey S. Diamond

**Affiliations:** 1 Synaptic Physiology Section, National Institute of Neurological Disorder and Stroke, National Institutes of Health, Bethesda, MD 20814, USA; 2 Department of Neuroscience, Brown University, Providence, RI 02912, USA; 3 Department of Ophthalmology and Visual Sciences, University of Wisconsin-Madison, Madison, WI 53705, USA; 4 Department of Neuroscience, University of Wisconsin-Madison, Madison, WI 53705, USA; 5 These authors contributed equally; 6 Lead contact

## Abstract

The neurovascular unit (NVU), comprising vascular, glial, and neural elements, supports the energetic demands of neural computation, but this aspect of the retina’s trilaminar vessel network is poorly understood. Only the innermost vessel layer—the superficial vascular plexus (SVP)—is associated with astrocytes, like brain capillaries, whereas radial Müller glia interact with vessels in the other layers. Using serial electron microscopic reconstructions from mouse and primate retina, we find that Müller processes cover capillaries in a tessellating pattern, mirroring the wrapping of brain capillaries by tiled astrocytic endfeet. Gaps in the Müller sheath, found mainly in the intermediate vascular plexus (IVP), permit diverse neuron types to contact pericytes and the endothelial cells directly. Pericyte somata are a favored target, often at spine-like structures with reduced or absent vascular basement lamina. Focal application of ATP to the vitreal surface evoked Ca^2+^ signals in Müller sheaths in all three vascular layers. Pharmacological experiments confirmed that Müller sheaths express purinergic receptors that, when activated, trigger intracellular Ca^2+^ signals that are amplified by inositol triphosphate (IP_3_)-controlled intracellular Ca^2+^ stores. When rod photoreceptors die in a mouse model of retinitis pigmentosa (*rd10*), Müller sheaths dissociate from the deep vascular plexus (DVP) but are largely unchanged within the IVP or SVP. Thus, Müller glia interact with retinal vessels in a laminar, compartmentalized manner: glial sheaths are virtually complete in the SVP but fenestrated in the IVP, permitting direct neurovascular contacts. In the DVP, the glial sheath is only modestly fenestrated and is vulnerable to photoreceptor degeneration.

## In brief

Grimes et al. use EM to examine the ultrastructure of neurovascular units in mouse retina and discover differences in glial coverage, neuronal contacts, and susceptibility to disease. Glial calcium signals are activated by ATP and propagate through all layers. Neurovascular contacts near photoreceptors are compromised in retinitis pigmentosa.

## Graphical Abstract

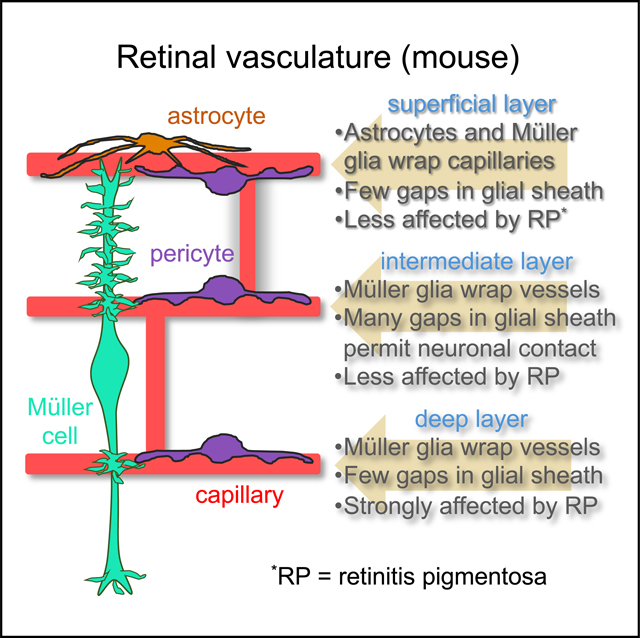

## INTRODUCTION

Retinal processing, like all neural computation, is metabolically expensive and requires extensive vascular support. Throughout the brain, pericytes, endothelial cells, and perivascular glia cooperate to (1) extract metabolic resources from the blood, (2) remove waste, (3) maintain the blood-brain barrier (BBB), and (4) dynamically control blood flow in response to changes in neural activity, a process known as neurovascular coupling (NVC) or functional hyperemia. Endothelial cells forming the capillary wall make tight junctions with one another, an essential element of the BBB. Endothelial cells are coated in basement lamina and partially covered by pericytes, contractile cells that cling to vessels’ external surface and are also surrounded by basement lamina.^1,2^

**Figure 1.**
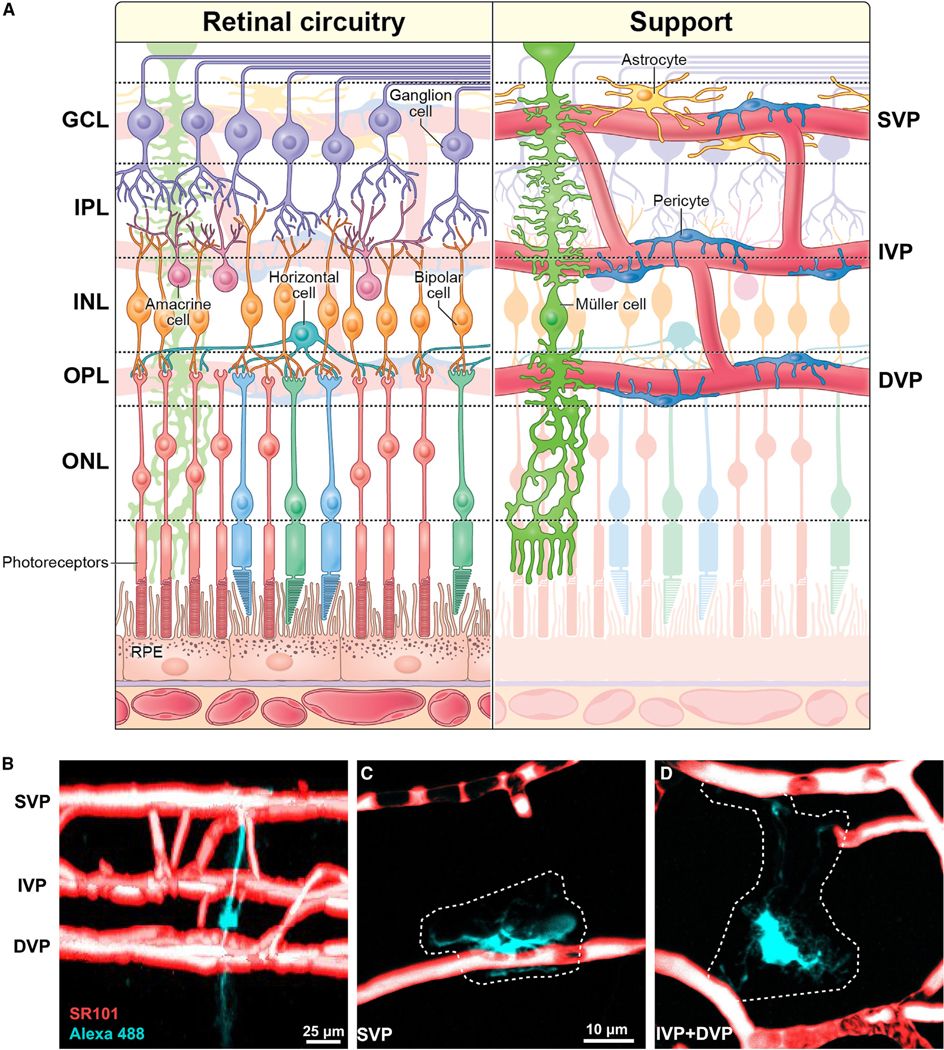
Organization of the mammalian retina (A) Left: schematic of retinal circuitry comprising five neuron classes. Right: schematic showing the retina’s support network of blood vessels, pericytes, and glial cells (see also Figure S1). GCL, ganglion cell layer; IPL, inner plexiform layer; INL, inner nuclear layer; OPL, outer plexiform layer; ONL, outer nuclear layer; RPE, retinal pigment epithelium; IVP, intermediate vascular plexus; DVP, deep vascular plexus. (B) Side (x-z) view of fluorescence micrograph showing the trilaminar vasculature (red, SR-101 pretreatment) and a Müller cell (teal, injected with Alexa 488). (C) *En face* (x-y) view at the level of the SVP. (D) *En face* (x-y) projection encompassing the IVP and DVP. See also Figure S1.

Most mammals, including humans, have highly vascularized retinas (Figure 1). Even brief interruptions in blood flow dramatically diminish retinal light responses (for review see Osborne et al.^3^). Although photoreceptors are nourished by the choroid, the rest of the retina receives its blood supply from the central retinal artery, which branches upon entering the eye to form a trilaminar vascular network comprising the superficial, intermediate, and deep vascular plexuses (SVP, IVP, and DVP; Figure 1). The SVP lies within the ganglion cell layer (GCL) and optic fiber layer and includes veins, arterioles, and capillaries.^4,5^ The IVP and DVP consist mainly of capillaries. The IVP is situated at the boundary between the inner nuclear layer (INL) and the inner plexiform layer (IPL), whereas the DVP lies in the outer plexiform layer (OPL) near the photoreceptor synaptic terminals.

In the central nervous system (CNS), capillaries and pericytes are ensheathed by glial endfeet. In the brain, the endfeet derive from astrocytes,^6^ but retinal astrocytes are confined to the SVP, where they, together with radial Müller glia, wrap vessels^7^ (Figures 1 and S1). In the IVP and DVP, only Müller glia wrap capillaries, as shown in cat,^8^ tree shrew,^9,10^ and human.^11^ Müller cells span nearly the entire depth of the retina, extending fine processes laterally in all synaptic and vascular layers.

Visual stimulation dilates blood vessels in all three vascular layers.^4^ In healthy animals, oxygen tension is lowest near the IVP,^12^ indicating high oxygen consumption relative to replenishment. The IVP also exhibits the largest relative changes in capillary diameter in response to visual stimulation,^4^ and local Ca^2+^ signals in Müller cells are linked to IVP capillary dilation,^13^ suggesting that the architecture of the IVP neurovascular unit (NVU) might differ from that in the SVP and DVP.

Here, we performed the first large-scale ultrastructural reconstructions of glial, neuronal, and vascular cells in the IVP and DVP using serial blockface scanning electron microscopy (SBFSEM). We found that Müller glia collectively provide near-complete ensheathment of these retinal capillaries in mice and non-human primates (>90% coverage). Nonetheless, gaps in the sheath, mostly in the IVP, allow pericytes and endothelial cells to make direct contact with bipolar, amacrine, and ganglion cells. Pericyte somas are a favored target of neuronal contacts, often at complex, spine-like appendages. Similar neurovascular architecture, including fenestrations in the glial sheaths permitting neuronal contact onto pericytes and endothelial cells, is apparent in neocortical SBFSEM datasets.

In live imaging experiments, exogenously applied ATP elicited Ca^2+^ signals throughout Müller cells, including the sheaths covering all three vascular layers. Pharmacological experiments suggested that Ca^2+^ signals reflect activation of Müller cell purinergic receptors that drive Ca^2+^ mobilization via inositol 1,4,5-trisphosphate receptors (IP_3_Rs). In early stages of retinal degeneration in *rd10* mice,^14^ we found that Müller sheaths were altered mostly in the DVP, near the photoreceptors.

Taken together, these findings provide new insights into the structural organization of the NVU in the mammalian retina. They identify novel interaction sites between neurons, pericytes, and endothelial cells and demonstrate that the NVU is disrupted in a layer-specific manner during retinal degeneration.

## RESULTS

### Near-complete ensheathment of retinal vessels by Müller glia

We examined publicly available three-dimensional (3D) SBFSEM datasets of C57/Bl6 mouse retina to visualize glial ensheathment of retinal vessels with high resolution. Our study builds upon earlier SEM analysis of astrocytic sheaths in the mouse SVP.^7^ We focused mainly on the IVP and DVP, where Müller cells rather than astrocytes wrap the capillaries, through intensive analysis of two mouse retina datasets.^15,16^ We also made more targeted observations in a third mouse retinal volume^17^ and a volume of macaque IPL (see STAR Methods). We used the Webknossos.org platform to reconstruct cells, mainly through manual tracings with skeletons and volume reconstructions of selected elements. Some volume reconstructions were also available with the archived Helmstaedter volume. In the Ding volume, identification of neuron class/type was aided by a large library of skeleton reconstructions, many of which have been described in previous publications^18–25^ (see STAR Methods).

We started with the Ding dataset, the largest of the mouse retinal datasets (Table S1). It spans the entire IVP, but SVP vessels are mostly cropped and DVP vessels are excluded completely. We combined skeletonization and volume annotation to produce 3D reconstructions of the vessels and the cellular processes that contact them. Müller cells were easily recognizable, even in single sections: most fine distal processes exhibited distinctively concave surfaces as they filled the spaces between the largely convex neuronal profiles. They also exhibited distinctively patterned intracellular organelles. We generated skeleton reconstructions of several neighboring Müller cells with single vertical stalks spanning the IPL and profuse, highly branched fine processes extending laterally for up to 15 μm in the IPL, often wrapping nearby IVP capillaries.

**Figure 2.**
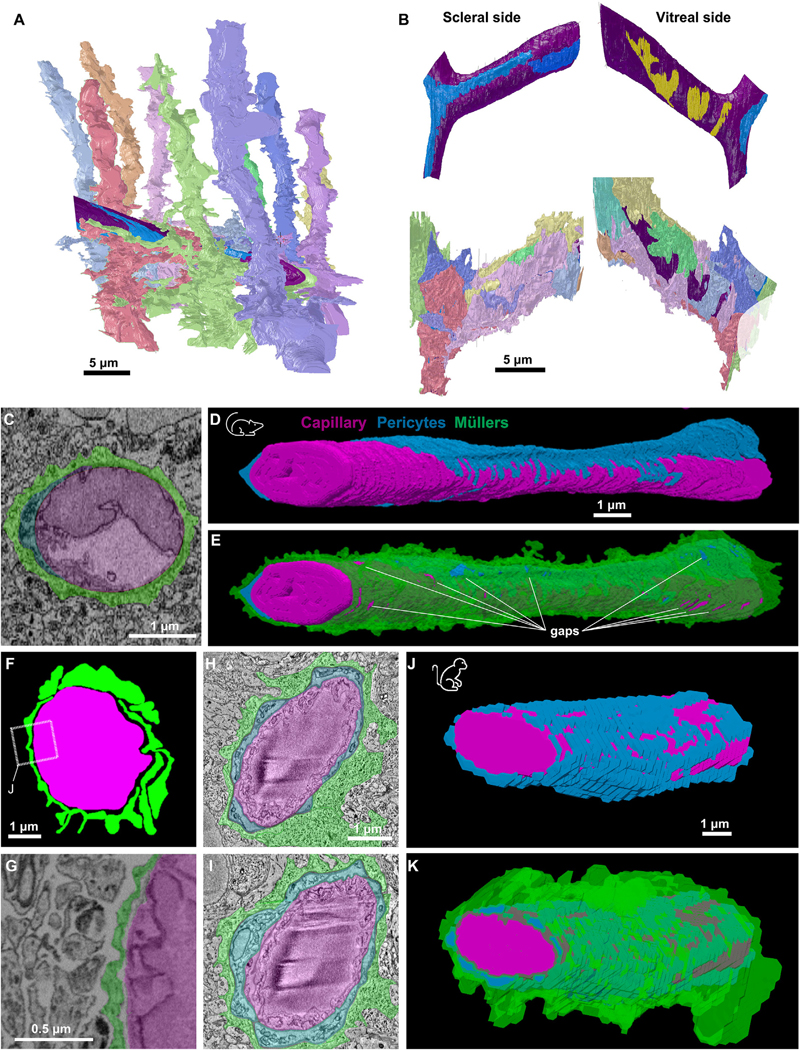
Müller sheaths around pericytes and capillaries in mouse and primate retinas (A) Volumetric reconstruction of Müller cells contacting vessel (purple) and an associated pericyte (blue). (B) Top: volumetric reconstruction of the IVP capillary (purple) and pericyte (blue) from (A). Gaps in Müller sheath indicated in yellow. Bottom: same views as above, but with Müller endfeet shown. Müller colors correspond to cells in (A). (C) Electron micrograph of a retinal capillary (magenta) in cross section, highlighting the anatomical arrangement with pericytes (blue) and Müller glia (green). (D) Volumetric reconstruction of an IVP capillary and the surrounding pericytes. (E) Same as in (D), with the addition of the Müller sheath. Same scale bar as in (D). (F) Colored image of a retinal capillary taken from an EM block in which extracellular space has been preserved.^17^ (G) Zoomed-in electron micrograph (from F) shows extracellular space between neurons and near-complete Müller sheath. (H–K) Ultrastructure of a retinal capillary from the IVP in a macaque (same color scheme as for other panels), similar to the arrangement found in mouse. (H and I) Capillary cross sections. Scale bar for (H) also applies to (I). (J and K) 3D reconstructions showing the IVP capillary wrapped by pericyte and Müller processes. Scale bar for (J) also applies to (K). See also Table S1.

We selected for detailed study a region containing a 20-μm stretch of a branching IVP capillary, making skeleton and volume reconstructions of the Müller processes contacting it, starting with the endfeet and tracing back through connecting processes to the main stalk. We also performed less-complete skeleton reconstructions to assess Müller-vascular relationships elsewhere in the volume (data not shown). We found that virtually all Müller glia contact IVP vessels passing within ~15 μm of their primary stalk. The vessel within the region of interest (ROI) was contacted by all ten of the Müller cells in this region of the mosaic (Figure 2A). Elsewhere in the volume, Müller shafts passing near an IVP vessel almost invariably extended at least one process to contact it. We did not observe contacts from Müller cells with stalks >15 μm from any IVP vessel.

Volume reconstructions (Figures 2A and 2B) indicated that two endothelial cells within this region formed the vessel walls and that two pericytes extended processes along the vessel surface. Numerous Müller endfeet contacted the capillary surface (Figures 2A and 2B). Individual Müller cells extended multiple endfeet that contacted the vessel, sometimes adjacent to one another but often surrounded by the endfeet of other Müller cells. The tessellated array of endfeet formed a sheath that was locally continuous and completely covered the scleral surface of the capillary, both endothelial cells and pericytes (Figure 2B). On the vitreal side, however, clear gaps appeared (see glimpses of purple endothelial surface between the pastel endfeet; Figure 2B bottom right, also shown as yellow patches in Figure 2B top right). Figure 2C shows a cross section of the IVP capillary, highlighting the extent to which Müller endfeet (green) encase the vessel. Reconstruction of another capillary section from the same dataset with the same color scheme (Figures 2D and 2E) also showed extensive Müller ensheathment with numerous gaps appearing throughout the sheath. As described in greater detail below, these gaps permitted direct contact between neurons and the vasculature.

We also analyzed a short segment (~5 mm) of IVP capillary in a mouse SBFSEM dataset in which fixation enhanced the extracellular space to highlight points of contact and adhesion between cells.^17^ A clear gap separated the Müller processes from the vascular wall, but the Müller processes themselves adhered to one another, forming a continuous glial sheath (Figures 2F and 2G), suggesting that the extensive Müller ensheathment observed in the Ding dataset is not an artifact of glial swelling due to chemical fixation.^26^

In a small SBFSEM volume from macaque retina, a reconstructed ~10 μm stretch of IVP capillary was completely covered by Müller endfeet that wrapped both pericytes and endothelial cells (Figures 2H–2K), indicating that extensive Müller glial coverage of IVP vasculature is conserved across the two species.

### Fenestrations in Müller sheaths permit direct interactions between neurons and IVP capillaries

**Figure 3.**
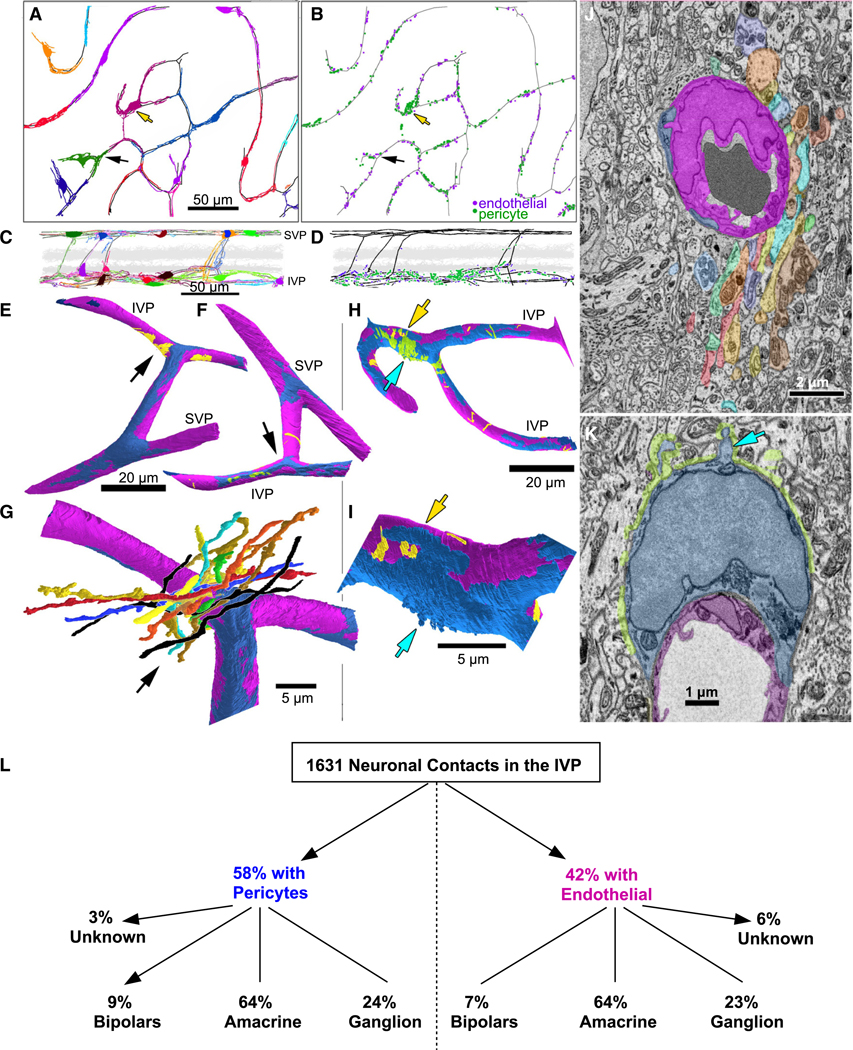
Direct contacts between neurons and vascular elements at Müller sheath gaps (A) Top-down (*en face*) views of the entire Ding mouse volume, showing IVP capillaries (black contours) and associated pericytes (colored skeletons). Linker vessels to the SVP are also shown (SVP vessels omitted for clarity). (B) Map of neuronal contacts onto the basement lamina of either endothelial cells (purple dots) or pericytes (green dots); black vessels and scale as in (A). (C and D) Vertical (side) views of the data in (A) and (B), respectively, except that the SVP and associated pericytes have been included (C). Scale in (C) applies to (D). (E and F) Volume reconstructions of endothelium (magenta) and pericytes (dark blue) in same region (see black arrows in A and B), showing neuronal contacts onto endothelial basement lamina (yellow) and pericyte basement lamina (green). All pericyte processes (except for darker blue, E, upper left) arise from one SVP pericyte soma (asterisk). (G) Enlarged view of subregion (E, black arrow) showing volume reconstructions cofasciculating neuronal processes making endothelial contact. Ganglion cell dendrites (black), amacrine processes (other colors). (H) Volume reconstructions of a different IVP region of the IVP (see yellow arrows in A and B) viewed from the vitreal side; conventions as in (E) and (F). Here, neurons contact primarily pericyte (green) rather than endothelium (yellow). Most contacts are onto the soma of the central pericyte (asterisk), which gives rise to all pericyte processes shown (except for darker blue, lower right). (I) Enlarged view of pericyte soma (asterisk as in H) exhibiting spine-like appendages (arrow). (J) Representative electron micrograph showing a slice through the region indicated by the arrow in (G), with same color scheme (e, endothelial cell; p, pericyte processes). (K) As in (J), but drawn from the region marked by the arrow in (H) and (I). Neuronal contacts onto pericyte soma (p*) are indicated in green. (L) Neuronal contacts broken down by vascular element and cell class. See also Table S1.

We systematically searched all vessels in the Ding volume for gaps in the glial sheath, where we saw that neurons contacted pericytes and endothelial cells (Figures 3A–3D). We made full skeleton reconstructions of all pericytes, which wrapped IVP vessels and IVP-SVP connector vessels with minimal overlap (Figures 3A and 3C). Neuronal contacts onto pericytes and endothelial cells (1,631 total contacts; Figure 3B) were restricted almost entirely to the IVP, with few contacts onto IVP-SVP connector vessels. 58% of the contacts were made onto pericytes, with the remainder onto endothelial cells (Figures 3B and 3D).

Volume reconstructions in this dataset (Figures 3E–3I) showed that patches of neuronal contact onto pericytes and endothelial cells ranged in size from a single localized contact to larger patches comprising contacts from more than a dozen neuronal processes in a tight fascicle (Figures 3G and 3J). Some neuronal contacts were found on peripheral pericyte processes (e.g., Figure 3F), but most were concentrated at or near the soma (Figure 3H). Numerous spine-like appendages protruded from the pericyte’s surface, increasing the available surface area for neuronal contacts (Figures 3F and 3K). Some neuronal processes contained small organelles resembling synaptic vesicles, but they did not cluster near contact points nor did we observe any other obvious structural features that would indicate presynaptic specializations.

All cells directly contacting pericytes and endothelial cells through gaps in the IVP Müller sheath appeared to be neurons. Some were not entirely contained within the dataset volume, but partial reconstructions typically provided enough information to determine cell class (see STAR Methods), although often not the specific cell type within that class. Of the 1,631 contacts, 49 could not be assigned to a class based on our criteria (Figure 3L). All three major neuronal classes within the IPL (i.e., bipolar, amacrine, and ganglion cells, identified as described in STAR Methods) made direct contact onto both pericytes and endothelial cells, with little difference in the proportion of contacts from each class (Figure 3L). Many distinct types, recognizable from their branching, stratification, and connectivity, contributed to each of these contacting classes. Together, they represented virtually all of the neuronal types with processes stratifying near the IVP,^16^ including A17, VIP, and dopaminergic amacrine cells and F-mini Off, JamB, suppressed-by-contrast, and OFF sustained alpha ganglion cells. Among bipolar cells, the OFF cone bipolar cells with axonal arbors closest to the IVP made most of the contacts, particularly types 1 and 2. Among all these cells, only the OFF bipolar cells exhibited an obvious bias toward pericytes. Though microglia have been shown to associate with and regulate vasculature in the retina and brain,^27,28^ none of six microglia that we reconstructed in the Ding volume contacted IVP capillaries.

**Figure 4.**
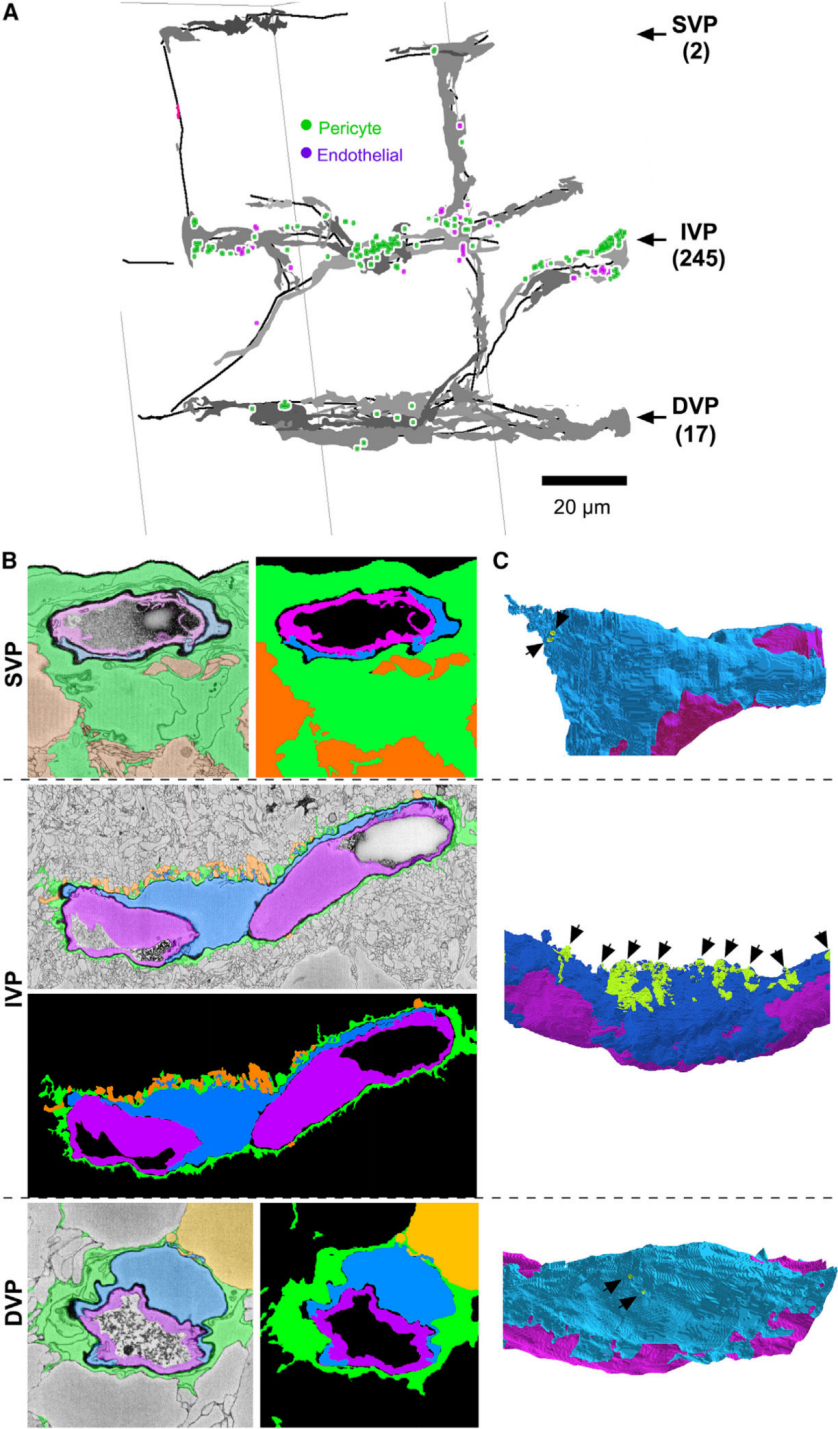
The IVP is more fenestrated than the SVP and DVP (A) Capillaries (black) and pericytes (gray) mapped for all three vascular layers in the Helmstaedter dataset, showing Müller sheath gaps over pericytes (green) and endothelium (magenta). Number of gaps indicated in parentheses. (B) Examples of near-complete Müller ensheathment in each vascular layer: Müller cells (green), pericytes (blue), and endothelial cells (magenta). (C) Partial 3D reconstructions of the capillaries and pericytes shown in (B). Exposed IVP pericyte somas often extend spines into the IPL (black arrows). See also Figures S2 and S3 and Table S1.

To examine glial sheaths in other layers, we performed similar reconstructions in the Helmstaedter dataset, which, though smaller than the Ding volume, includes all three vascular plexuses.^29^ The SVP was covered almost completely by Müller and astrocytic glial processes, as recently reported,^7^ although we did identify two small gaps where a vessel was contacted by ganglion cell axons (Figures 4A–4C top and S2). Gaps were more common in the IVP (*n* = 245) than in the DVP (*n* = 17). As in the Ding volume, many of the gaps were associated with spiny protrusions from the pericytes, concentrated near the soma (Figure 4C middle). The basement lamina, particularly prominent in this dataset, was thinner around these spines (Figure S2). Similar spine-like appendages in endothelial cells also received neuronal contacts. We could not assess possible synaptic specializations in this volume, which had been prepared specifically to mask intracellular detail.

### Fenestration of the glial sheath also permits neuron-to-vessel contacts in neocortex

To determine whether other CNS capillaries might exhibit similar fenestration of the glial sheath, we analyzed capillaries in two publicly available SBFSEM datasets of mouse somatosensory cortex.^30,31^ We found many instances in both where neuronal profiles penetrated the astrocytic sheath to contact endothelial cells as well as pericytes. In a P5 mouse (layers 2–4), neuronal contacts were scattered widely across the pericyte’s surface (Figure S3). Many contacts were made onto endothelial spines, some of which penetrated the glial sheath to contact neurons (Figures S3G–S3K). Local reconstructions of the contacting neurons showed that, as in the retina, they were diverse. Contacts were made by dendritic spines, axonal varicosities, and some neuronal somas (Figures S3J and S3K). These findings, including the diversity of contacting neuronal types, were confirmed in a volume acquired from layer 4 of somatosensory cortex from a P28 mouse.

### ATP-evoked Ca^2+^ activity in Müller sheaths

**Figure 5.**
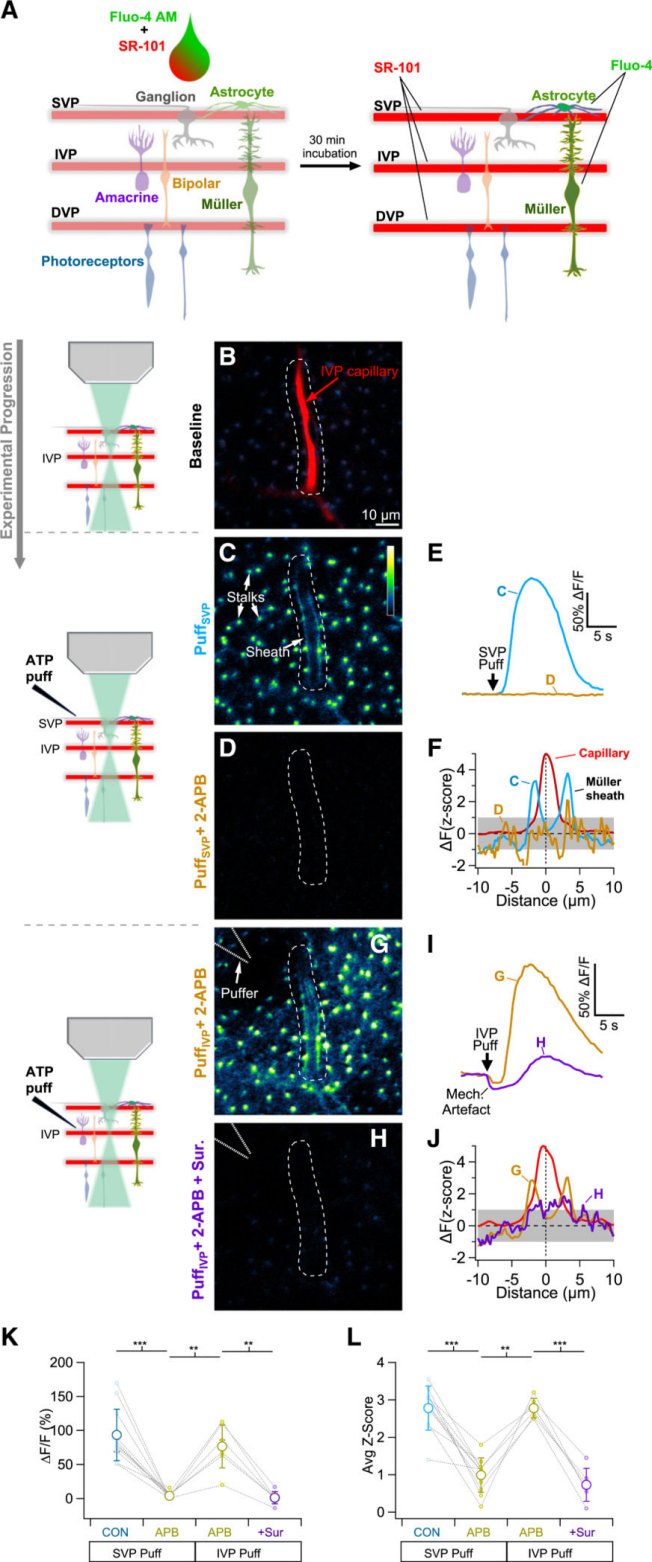
Purinergic Ca^2+^ signaling in Müller sheaths (A) Schematic showing vitreal application of Fluo-4 AM and SR-101 to load glia and blood vessels, respectively. See also Figure S4. (B) SR-101-loaded IVP capillary (~50 μm below the vitreal surface). The same field of view and region of interest (dotted line) apply to subsequent images and measurements. (C) Baseline-subtracted Fluo-4 (Ca^2+^) signals in peri-IVP capillary Müller glial processes evoked by ATP puff application at the vitreal surface (Puff_SVP_). See also Figure S5. (D) IVP Fluo-4 signals in response to Puff_SVP_ are blocked by the IP_3_R antagonist 2-APB (100 μM). (E) Puff_SVP_-evoked Fluo-4 responses. (F) Puff_SVP_-evoked Fluo-4 profiles of capillary sheaths in control (blue) and in 2-APB (gold). Capillary signal (red) is normalized to a *Z* score = 5. Gray region indicates ±1 standard deviation. (G) Baseline-subtracted Fluo-4 signals evoked by ATP puffed directly onto an IVP capillary (Puff_IVP_) in the presence of 2-APB. (H) As in (G), in the additional presence of the purinergic receptor antagonist suramin. (I) Puff_IVP_-evoked Fluo-4 responses. (J) Puff_IVP_-evoked Fluo-4 profiles of capillary sheaths in 2-APB ± suramin. Analysis as in (F). (K and L) Population data showing Fluo-4 responses (K) and average *Z* score (L) of each vessel under each experimental condition. Error bars indicate standard deviation. ** = *p* < 0.01, *** = *p* < 0.001.

Ca^2+^ signals in Müller glia play an important role in IVP capillary dilation,^13^ and previous work has shown that extracellular ATP elicits robust Ca^2+^ signals in Müller glia,^32,33^ but less is known about Ca^2+^ activity within the capillary sheaths. To address this, we labeled blood vessels with sulforhodamine (SR101) and loaded Müller cells (and astrocytes) with the fluorescent Ca^2+^ indicator Fluo-4-AM (Figure 5A, see STAR Methods) to monitor Müller cell Ca^2+^ responses to brief puffs of ATP (Figures 5C–5L). Fluo-4-AM was applied to the vitreal surface, which allowed it to enter astrocytes and Müller endfeet, but the inner limiting membrane (ILM) largely prevented the dye from entering neurons (Figure S5).^34^ Consistent with previous work showing that Müller glia are highly sensitive to extracellular ATP,^32,33^ we found that briefly puffing 5 mM ATP onto the retina’s ILM elicited Ca^2+^ signals in Müller cells at all retinal depths, including the sheaths surrounding IVP and DVP capillaries (Figures 5B, 5C, and S5; see STAR Methods). We quantified these signals in two ways: (1) a gross measurement of Müller Fluo-4 signal in the vicinity of capillaries (i.e., ΔF/F of an ROI extending ~10 μm in each direction from the capillary center) to account for loading or responsivity issues (Figure 5E) and (2) a normalized measure of sheath Ca^2+^ signaling and morphology relative to the capillary (ΔFZ score, Figure 5F; see STAR Methods).

Bath application of the IP_3_R antagonist 2-APB reduced Fluo-4 responses in IVP Müller sheaths to brief ATP puffs (50 ms) at the retinal surface ($\text{ΔF}/{\text{F}}_{2-\text{APB}}=6\%\pm 9\%$ of control, $Z\;{\text{score}}_{2-\text{APB}}=38\%\pm 21\%$ of control, *n* = 11 capillaries, Figures 5C–5F). In the continued presence of 2-APB, the puff electrode was then moved into the IPL to puff ATP directly on the capillary of interest. Direct puffs elicited robust Fluo-4 responses in ensheathing Müller processes, even in the presence of 2-APB (*Z* score_2-APB_: superficial puff = 0.95 ± 0.52 vs. intermediate puff = 2.7 ± 0.2, *n* = 6, Figure 5G, 5I, and 5K). These responses were blocked by the purinergic receptor antagonist suramin (*Z* score = 26% ± 13% of 2-APB alone, ΔF/F=−8%±26% of 2-APB alone, *n* = 6, Figures 5H–5L). These results indicate that the Müller processes that ensheathe capillaries express purinergic receptors and exhibit Ca^2+^ responses to ATP that are propagated by IP_3_R-mediated release from intracellular stores.

### Disruption of sheath activity in a mouse model of retinal degeneration

**Figure 6.**
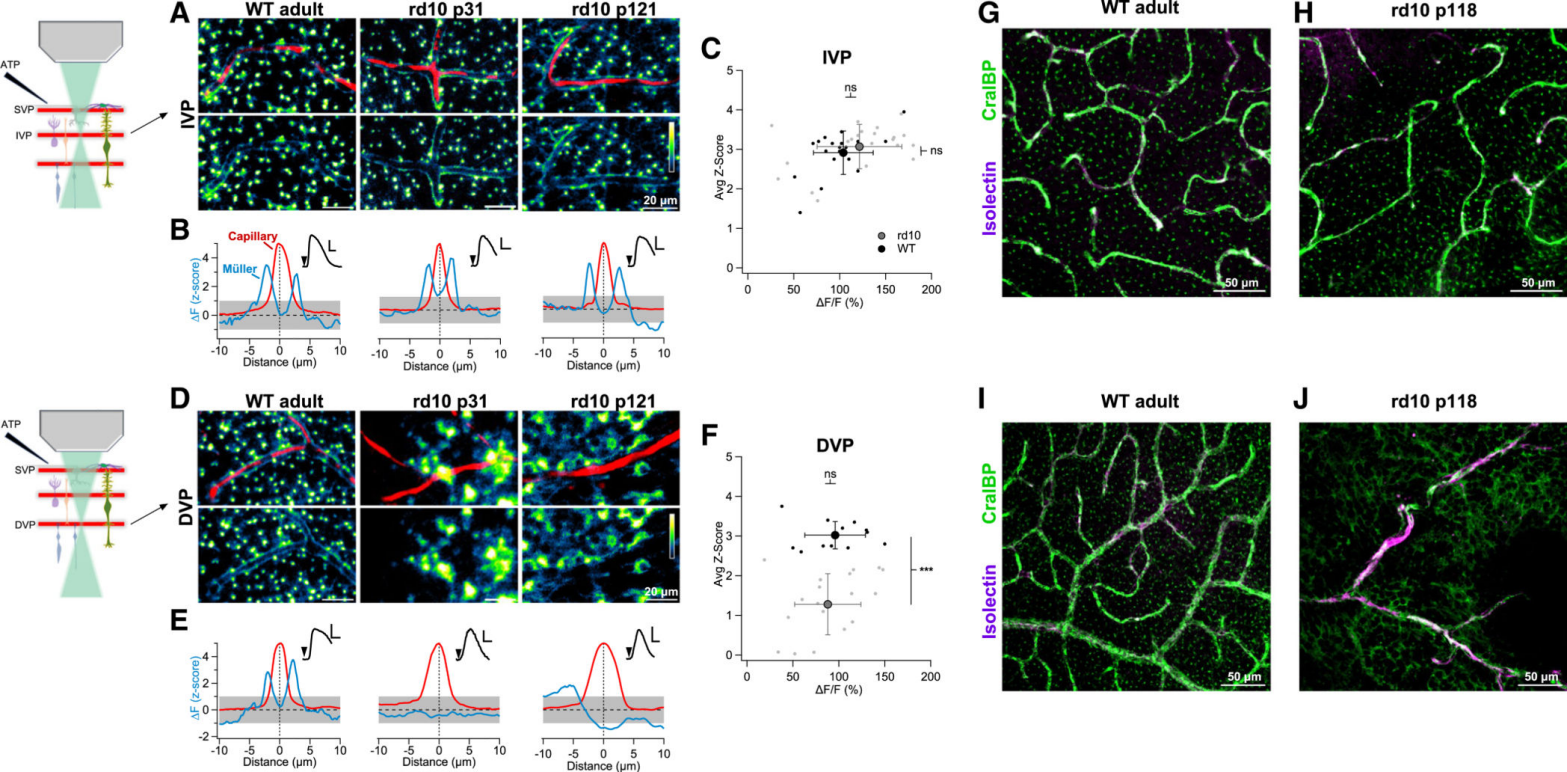
Müller sheaths are perturbed during retinal degeneration (A) Baseline-subtracted Müller cell Fluo-4 ΔF signals (green) in the IVP evoked by a superficial ATP puff in WT and rd10 retinas. Capillaries are labeled with SR101 (red). (B) ΔFZ score (blue) and capillary profile (red, normalized to 5) vs. distance from the center of the capillary. Insets ΔF/F Fluo-4 signals evoked by brief ATP puffs. (C) Population data for sheath activity in the IVP. Each point represents one vessel segment (≥1 mm of capillary was analyzed for each condition). Larger symbols with error bars indicate mean ± standard deviation. (D–F) As in (A)–(C) but for DVP capillaries. *** = *p* < 0.001. (G–J) Immunohistochemical assessment of vascular and Müller cell morphology in WT vs. rd10 retina. Müller glia are labeled with CralBP (green), capillaries with isolectin (magenta). (G and H) Comparable labeling patterns were observed in the IVP of WT (G) and rd10 (H) retinas. (I and J) In the DVP, total capillary length was reduced and Müller morphology was disrupted in rd10 (J) compared with WT (I).

Many retinal diseases, including retinitis pigmentosa and diabetic retinopathy, trigger gliosis.^35^ To examine how these pathological processes affect Ca^2+^ signaling in Müller sheaths and their association with retinal capillaries, we repeated our imaging experiments in the *rd10* mouse model of retinitis pigmentosa (Figure 6). *rd10* mice have a missense protein mutation in rod photoreceptors that leads to their early death; as rods die, gliosis is triggered and the retina begins to remodel.^35^ Wild-type (WT) and *rd10* retinas were loaded identically with Fluo-4 AM and SR101 and imaged while delivering ATP puffs on the retinal surface. This stimulus evoked robust Fluo-4 signals in Müller processes in the IVP in both WT (*Z* score = 2.9 ± 0.5, *n* = 19) and *rd10* mice (*Z* score = 3.1 ± 0.6, *n* = 21) (Figures 6A–6C). In the DVP near rod synaptic terminals, however, we detected clear differences in the relationship between Müller sheath activity and capillaries. In WT mice, capillaries in the DVP were surrounded closely by a thin rim of Ca^2+^ activity (*Z* score = 3 ± 0.3, *n* = 12, Figures 6D–6F). In *rd10* mice, however, Müller processes in the deep capillary layer were more diffuse and irregular. Although *rd10* Müller glia took up Fluo-4 and responded to ATP puffs (ΔF/F=88%±36%), these indicator signals were no longer tightly associated with the vessels (*Z* score = 1.3 ± 0.8, *n* = 19, Figures 6D–6F).

To determine whether altered Ca^2+^ signals in the DVP of *rd10* retinas reflect changes in Müller cell morphology, we labeled capillaries (with isolectin) and Müller glia (with CralBP) in WT and *rd10* retinas (Figures 6G–6J). These experiments revealed only minor changes in Müller morphology within the IVP of *rd10* and extensive morphological changes within the DVP. Together, these results indicate that photoreceptor degeneration disrupts Müller sheaths locally and in a layer-specific manner.

## DISCUSSION

**Figure 7.**
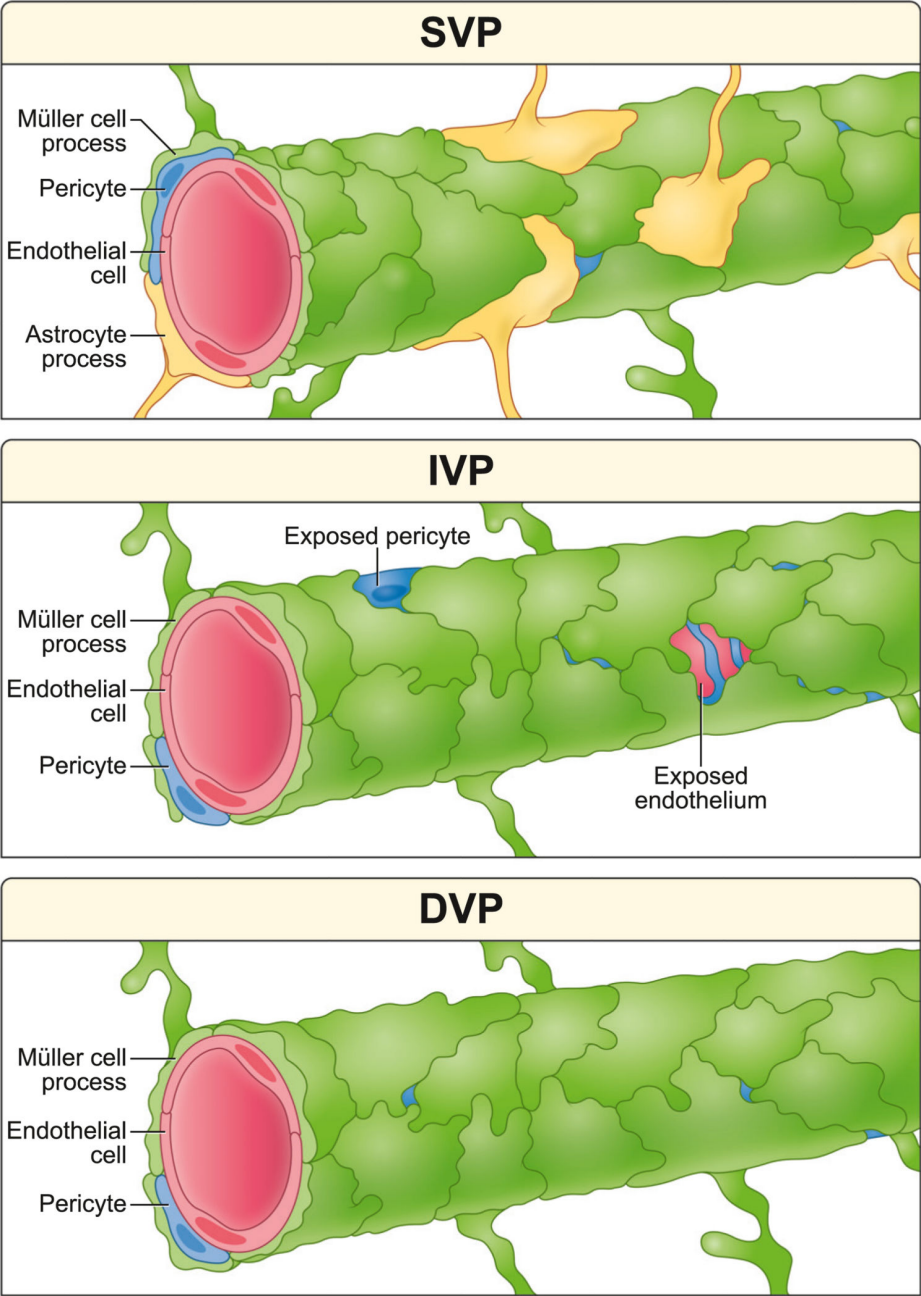
Illustrated conclusions from this study Top: SVP capillaries are wrapped by astrocytes and Müller cells. Sheaths are essentially complete, limiting opportunities for neuronal contact. Middle: Müller glia cover IVP capillaries with fenestrated sheaths that allow neurons to directly contact endothelial cells and pericytes. Bottom: DVP capillaries are ensheathed by Müller glia with few gaps.

The retina’s vasculature supports the metabolic demands of visual processing. Here, we show that Müller glia encase capillaries in all three vascular layers of the mouse retina (Figure 7). Though these sheaths are nearly complete, gaps exist, especially in the IVP, allowing direct neurovascular contacts. When gliosis is triggered by photoreceptor degeneration, the Müller sheath is disrupted primarily in the DVP.

### Neuronal interactions with the perivascular elements

The near-complete (>90%) Müller glial coverage that we observe around retinal capillaries parallels capillary anatomy in the hippocampus—where the glial sheath is formed by astrocytic endfeet^6,27^—and our preliminary analysis of the neocortex (Figure S3). Müller processes forming the sheath appear to adhere tightly to one another (Figure 2), suggesting that glial sheaths insulate vessels from the surrounding milieu, permitting interactions between neurons and vessels only at sparsely distributed gaps. Neurons contact both endothelial cells and pericytes, apparently favoring pericyte somata. The contacting neurons appear to represent most of the neuronal types present near the gap. Interestingly, the balance and ultrastructure of contacts made onto endothelial cells or pericytes appear to vary in different brain regions. For example, hippocampal endothelial cells are almost entirely wrapped by glia, with only a few small gaps allowing contacts with presumed microglial processes,^27^ whereas pericytes get more extensive neuronal contacts. In the retina and somatosensory cortex, we observed a more equal balance of endothelial and pericyte contacts. In the neocortex, we found many neuronal contacts at tight junctions between endothelial cells that contribute to the BBB. In the IVP of the retina, many neuronal contacts were made onto pericyte somatic spines. These were far less common in the cortical volumes studied, though at least one reconstructed endothelial cell extended a surprisingly long spine into the neuropil, where it was covered in neuronal contacts. In the embryonic neocortex, endothelial cells exhibit striking spinous appendages, some penetrating processes of neighboring neural progenitor cells.^36^

The functional importance of these diverse neurovascular contacts remains unclear. Physiological results suggest that Müller glia, like astrocytes, play an intermediary role in NVC.^13^ Is there a role for the direct neural contacts reported here? These contacts are most prevalent in the IVP, which exhibits the largest activity-dependent changes in vessel diameter.^4^ Neurovascular contacts are made by diverse types of bipolar, amacrine, and ganglion cells extending processes at the level of the IVP. These contacts lacked obvious presynaptic specializations, suggesting that these neurons may not use vesicular synaptic transmission to communicate with perivascular elements. A precedent for direct neurovascular communication exists in the glutamatergic signaling to smooth muscle cells in neocortical arterioles.^37^

Other signaling mechanisms may be involved, including gap junctions, transporter-mediated signaling, and membrane-permeant molecules like nitric oxide. It is also possible that signals pass in the opposite direction, from vessels to neurons and glia, along with glucose and oxygen. Molecular cues in the bloodstream modify neural stem cell activity during development and adulthood: specifically, in mouse neocortex blood, cues help convert a single layer of proliferating progenitor cells into multiple distinct neuronal layers.^38^ Given that Müller cells act as radial glial late-stage progenitors under certain conditions, cues in the blood may guide cell differentiation after injury.^39–41^

We observed marked variability in glial coverage of capillaries across and within the different brain regions examined. In the mouse datasets, neurovascular contacts were most abundant in the IVP, though this requires confirmation in other volumes. Glial coverage also appears to vary during development: in the cortex, many contacts were observed in P5 mouse somatosensory cortex but fewer at P28. A survey of other cortical datasets (Webknossos.org; MiCRONS project primary visual cortex) revealed many capillary segments with few if any neuronal contacts, similar to the virtually complete ensheathment of hippocampal endothelial cells^6^ and the short stretch of IVP capillary we analyzed from macaque retina. It is unclear what accounts for this diversity in neurovascular contacts; species, age, laminar location, and vessel branch order may all be factors.

### Müller sheaths and disease

Many neural diseases trigger gliosis, i.e., glial activation and/or proliferation. Here, we examined Müller activity in the IVP and DVP in the *rd10* mouse model of retinal degeneration. Previous work in *rd10* mice showed that, as rod photoreceptors die, the blood-retinal barrier (BRB) breaks down first in the DVP, followed by the IVP, and eventually the SVP.^14^ Our results from *rd10* mice (p31–p121), showing layer-specific alterations in Müller sheaths, are consistent with this study, suggesting that barrier disruption coincides with loss of Müller ensheathment. Because degeneration in the *rd10* mouse model originates within rod photoreceptors, Müller processes in the deep layers may sense cell death and alter their function, as shown in P23H mice, another model of retinitis pigmentosa.^42^ If Müller cells adapt to support dying photoreceptors, important resources might be diverted from BRB maintenance. Our data showing that sheath disruption, like BRB breakdown, is layer-specific, suggest that alterations in Müller morphology might be highly compartmentalized. Future experiments may determine how Müller sheaths react in other diseases, such as diabetic retinopathy, where external factors in the blood (i.e., glucose) facilitate a global influence on the BRB.

## RESOURCE AVAILABILITY

### Lead contact

Further information and requests for resources should be directed to and will be fulfilled by the lead contact, Jeffrey S. Diamond (diamondj@ninds.nih.gov).

### Materials availability

This study did not generate new, unique reagents.

### Data and code availability

- Original electrophysiology data have been deposited at Mendeley Data (https://data.mendeley.com/datasets/g63c5ypwgd/1) and are publicly available as of the date of publication. The DOI is listed in the key resources table. Microscopy data reported in this paper will be shared by the lead contact upon request.
- This paper does not report original code.
- Any additional information required to reanalyze the data reported in this paper is available from the lead contact upon request.

## STAR★METHODS

### EXPERIMENTAL MODEL AND STUDY PARTICIPANT DETAILS

We used wild type and rd10 mice of either sex for our experiments, ranging in age from p31-p121. Primate retinal tissue was obtained from an adult male *Macaca nemestrina* (macaque) through the Washington National Primate Center Tissue Distribution Program. All procedures were approved by the University of Washington, University of Wisconsin-Madison and/or NINDS Institutional Animal Care and Use Committee (ASP-1344).

### METHOD DETAILS

#### Sources of mouse and macaque retinal and cortical SBFSEM datasets

Mouse retinal and cortical SBFSEM datasets have been described in detail^15–17,30,31^ (Table S1). The published EM image data were accessed mainly through Webknossos.org, which hosts these data and provides tools for annotation and reconstruction. The Pallotto volume was provided by the authors via datadryad.org and analyzed in Webknossos.

The Ding volume has a voxel size of 13.2 × 13.2 × 26 nm,^15^ a resolution that permitted vesicles and chemical synapses to be visualized, but not gap junctions. Though having the same voxel size as the Ding dataset, the Pallotto volume was prepared specifically to preserve extracellular space, thereby enabling electrical synapses to be identified.^17^ The Helmstaedter block (16.5 × 16.5 × 26 nm voxel size) was prepared using methods to highlight extracellular spaces and minimize contrast of intracellular structures to facilitate reconstructions.^16^ The Motta block (11.24 × 11.24 × 28 nm voxel size) was drawn from Layer 4 of adult mouse somatosensory cortex.^31^ The Gour block (11 × 11 × 30 nm voxel size) was taken from P5 mouse somatosensory cortex.^30^

Part of the macaque volume has been published.^43^ In short, the macaque volume was prepared by dissecting the living retina in bicarbonate buffered Ames solution. A piece was cut from peripheral retina and immersion fixed in 4% glutaraldehyde in 0.1 M sodium cacodylate buffer (pH 7.4). The fixed sample was rinsed in cacodylate buffer and processed for serial block face scanning electron microscopy following an established protocol.^44^ Sectioning and imaging were performed on a 3View (Zeiss) serial block face scanning electron microscope at the University of Wisconsin-Madison School of Medicine electron microscopy facility. Images were acquired at 5 × 5 × 50 nm voxel resolution. Multi-montage acquisition was used to capture a wide field of view across the retina with each tile spanning ~45 μm. Sectioning and imaging was performed across the vertical cross-section.

#### Analysis of SBFSEM data

We used both skeletons (“line drawings” of neurons, with connected segments representing branching processes) and volume reconstructions (more complete renderings of the entire cell surface) in this study. Skeletons were generated within WebKnossos by placing a first node within a cell profile in a single serial section and adding additional connected nodes, each linked to the next, in subsequent sections along the interior of that process. Branchpoints were marked as they were encountered for later completion of the missing branches. Nodes could be placed sparsely (i.e., it is not necessary to place a node in every serial section) while still permitting a sketch of the cell’s overall shape. Skeletons were manually generated much more rapidly than volume reconstructions: For example, the extent of a single Müller cell shown in Figure 2A could be skeletonized in ~20 min, whereas manual volume reconstruction of that same segment took roughly ten times as long. Though they do not describe the cell’s surface, skeletons can contain specified nodes, located at sites of interest (such as vascular contact points), that can be labeled and managed separately. Relatively complete skeleton reconstructions of single cells provide valuable three-dimensional data about branching structure, stratification and synaptic connectivity, permitting identification of neuronal class and cell type. Volume reconstructions are formed by defining the cross-sectional profile of a cell of interest in each image plane that contains it. Typically, this is done by ‘painting’ the relevant area with a unique marker for that cell in each serial section, though Webknossos.org has developed tools to accelerate the process, for example by automatically interpolating the spatial connection between manually painted profiles in adjacent sections. Even with these advantages, volume reconstructions are much more labor-intensive to produce than skeletons, but they can provide more information about the spatial relationships among the membrane surfaces of different cells. Further details about analytic strategies for serial EM data can be found at WebKnossos.org (and see Cocks et al.^45^).

Most of the new reconstructions reported here were performed by a single annotator (A.S. or D.B.); those in the mouse retina and cortex were either generated by or checked by an expert (D.B.). Reconstructions are vulnerable to misleading false divisions or unions, although such errors would have a relatively minor impact on our core conclusions about Müller cell coverage of the vasculature. It is difficult to estimate reconstruction accuracy without “ground truth” data or multiple redundant reconstructions by many annotators,^16,46^ resources that were unavailable for the current work. The reconstructions generated here have been added to a growing catalog of annotated cells in the data sets, enabling interested readers to examine (and build upon) our annotations using freely accessible web-based resources (e.g., https://weblium.webknossos.org/).

We used several different annotation and visualization platforms for the reconstructions illustrated in this report. For the Ding volume, we used both the standalone Knossos program (https://knossos.app/) and the Webknossos.org web-based application to make skeleton and volume reconstructions. Reconstructions in cortical volumes were all generated in Webknossos, while the Pallotto dataset was reconstructed only in Knossos (https://knossos.app/). For the macaque block, reconstructions and annotations were performed using ImageJ (http://imagej.nih.gov), with renderings of skeletons and volumes generated using Paraview.^47^ Some skeleton and 3D volumetric reconstructions generated in Webknossos were exported for volume renderings in Blender.

To determine which cell types contact capillaries, we relied where possible on available cell reconstructions. For the Ding volume, we drew upon an extensive library of skeletons encompassing diverse types of neurons but also other retinal cell types (e.g., microglia; vascular). These have formed the basis of several prior reports on a diverse collection of cell types and neural circuits.^18–25^ The link to the Ding volume loads a master annotation file that includes these published skeletons as well as new ones generated for this report. Webknossos.org also provides extensive proofread segmentation for the Helmstaedter and Motta volumes, which helped us identify cell class or type.

Both the shapes of partially reconstructed cells and their distinctive intracellular features were used to identify specific cell types. For most profiles, it was possible to infer cell class (if not type) from examination of local features near the locus of vascular contact. Capillaries were readily identified at low magnification from their hollow profile bounded by a continuous ring of endothelial cells. Pericytes were identified by their close association with outer surfaces of endothelial cells. Their somas and long flattened processes clung to the vessels, exhibiting characteristic peg-and-socket arrangements of their fine terminal processes with endothelial and glial cells. Müller glia were recognizable from their stout stalks, concave space-filled processes, as well as their abundance of small dark organelles (~10 nm diameter; probably smooth endoplasmic reticulum). Microglia had a distinctive ultrastructure, with small somas, small mitochondria, prominent smooth endoplasmic reticula, and finely branched processes that look ‘blank’ due to the lower density of intracellular organelles. Bipolar cells were identified by the presence of synaptic ribbons.

#### Ca^2+^ imaging

Prior to experiments, mice were deeply anaesthetized with isoflurane (Baxter) and euthanized vis cervical dislocation. Once euthanized the animals were enucleated and the eyes were submerged in Ames medium (room temperature, continuously bubbled with 95% O2/5% CO2 gas). A small incision was made at the limbus, and scissors we then used to remove the cornea. The lens and vitreous were removed with forceps, and tissue was stored for up to 5 hours in this condition. Individual pieces of retina were isolated from the pigment epithelium and placed on a 10mm coverslip coated with poly-l-lysine as needed. The edge of a Kimwipe was used to remove excess solution prior to application of 200 μL of freshly gassed Ames, containing 62.5 μM Fluo-4 AM and 1 mM SR101, directly to the top of the wicked-down retina. The coverslip was mounted in a recording chamber and placed in a dark incubator with continuous carbogen flow for 30 min. After incubation the recording chamber was placed under the microscope and superfused with freshly gassed Ames (~8 mL/min) for at least 10 minutes before collecting data. Ca^2+^ signals and blood vessels were imaged with a Zeiss LSM510 confocal microscope (λ=488 nm). A 8–10 MU glass electrode filled with HEPES-buffered Ames containing 5 mM ATP was used to puff (Picospritzer, 5 psi, 0.05–1 s in duration) ATP on the retinal surface (i.e. ILM) or directly onto the intermediate capillaries. For reference, we estimate that the IVP and DVP are ~50 and ~90 microns below the retinal surface, respectively.

#### Immunohistochemistry

Dissected Retina was post-fixed containing 4% Paraformaldehyde (PFA) in 1x PBS for 30 minutes at RT, rinsed with PBS and store in 4 °C until use. Stored retinas then were blocked for 24 hours in a PBS solution plus 0.5% Triton X-100 and containing 10% normal donkey serum (NDS). Primary antibodies were diluted in the same solution and applied for 72–96 hours, followed by incubation for 24 h in the appropriate secondary antibodies. In Figure S1, we used GFAP (Sigma, 1:1000) and CD31 (R&D, 1:1000) to label astrocytes and blood vessels, respectively. In Figure 6, we used CralBP (Abcam, 1:200 and Isolectin (ThermoFischer, 1:50) to label Müller glia and capillaries, respectively. All steps were completed at in 4°C.

After staining, the tissue was flat-mounted on a slide, ganglion cell layer up, and cover slipped using Vectashield mounting medium (H-1000, Vector Laboratories). Immunoreactivity was visualized and acquired with a confocal microscope (Zeiss LSM780) using 20x air or 63×/1.4 oil objectives. The selected images were cropped and aligned with Fiji (https://imagej.net/software/fiji/).

### QUANTIFICATION AND STATISTICAL ANALYSIS

For Ca^2+^ imaging experiments, time series were analyzed in Fiji, and group data were compiled in Igor Pro. We focused primarily on capillaries in the intermediate and deep layers, and only analyzed sections that were fully within the plane of focus. Brief ATP puffs evoked radially expanding waves of Ca^2+^ activity, and analysis was also limited to within these regions. From these movies two measurements were extracted: 1) $\text{ΔF}/{\text{F}}_{0}$ for a ROI that extended approximately three vessel diameters in both directions from the center of the capillary of interest—as a general readout of puff-evoked Ca^2+^ signals in Müller glia (Equation 1):

(Equation 1) $$\frac{\text{Δ}F}{F_{0}}=\frac{F-F_{0}}{F_{0}}$$

and 2) a vessel-centric 2D analysis of the structure of local Ca^2+^ signals. For the vessel-centric 2D analysis 8–10 movies frames were averaged prior to stimulation and at the peak of the puff-evoked Ca^2+^ signal. The averaged baseline image was then subtracted from the averaged stimulation image to isolate the puff-evoked Ca^2+^ response. Ca^2+^ signals attributed to Müller stalks were then digitally removed from the baseline-subtracted image by: 1) thresholding the top 2–4% of pixel values of the averaged peak image (i.e. not baseline subtracted), 2) converting the thresholded pixels into a mask, 3) dilating the binary mask two-three times and 4) applying the mask to the baseline-subtracted image. This process converted stalk pixels to NaN which were then excluded from analysis. The resultant image was then combined with the SR101 labelling of blood vessels. Capillaries with curvature in the plane of focus were straightened with Fiji’s *straighten* function before taking vessel-centric measurements. To quantify the strength and locality of puff-evoked sheath activity we examined fluorescence signals as they extend from the center of the vessel. Average fluorescence signals were collapsed along the vessel axis for the entire length of the in-focus capillary of interest, and to a minimum of 20 μm on either side of the vessel. These profiles were converted to z-scores (Equation 2), and we quantified the average peak fluorescence from both sides adjacent to the vessel (Equation 3). Fluorescence originating from the blood vessels was normalized to a z-score of 5 for easy viewing.


(Equation 2) $$z-score=\frac{\text{Δ}F}{\sigma }$$

(Equation 3) $$ave.\;z-score_{peaks}=\frac{left\;z-score_{peak}+right\;z-score_{peak}}{2}$$

All statistical analysis was performed using Igor Pro. Reported n values indicate number of cells. Normally distributed (as determined by the Jarque-Bera test) group data values are reported as mean ± SD and compared with a *t*-test. Significant differences were concluded if *p* < .05.

## Supplementary Material

mmc1

## Figures and Tables

**Table:** KEY RESOURCES TABLE

REAGENT or RESOURCE	SOURCE	IDENTIFIER
Antibodies		

GFAP (mouse polyclonal)	Sigma	G3893; RRID:AB_477010
CD31 (goat polyclonal)	R&D	AF3628; RRID:AB_2161028
Isolectin-Alexa Fluor conjugate	ThermoFisher	I21411; AB_2314662
CralBP (mouse monoclonal)	Abcam	AB15051; RRID:AB_2269474

Chemicals, peptides, and recombinant proteins		

Ames media	US Biological Life Sciences	A1372-25
NaHCO_3_	Fisher Bioreagents	BP328-500
Mg ATP	Millipore Sigma	A9187
AlexaFluor 488	ThermoFisher	A10436
Sulforhodamine-101	Millipore Sigma	S7635
HEPES	Millipore Sigma	H3375
Suramin	Millipore Sigma	S2671
2APB	Tocris Bioscience	1224
Triton X-100	Millipore Sigma	9036-19-5

Deposited data		

Igor files	Mendeley Data	Mendeley Data: https://data.mendeley.com/datasets/g63c5ypwgd/1

Experimental models: Organisms/strains		

mouse: wild-type: C57BL/6J	Jackson Laboratory	000664; RRID:IMSR_JAX:000664
mouse: Rd10	Jackson Laboratory	004297; RRID:IMSR_JAX:004297
primate: Macaca nemestrina	Washington National Primate Center Tissue Distribution Program	RRID:SCR_002761

Software and algorithms		

Stage	Github	https://github.com/Stage-VSS/stage
Symphony	Github	https://github.com/Symphony-DAS/symphony-matlab
Data Analysis package	Github	https://github.com/Schwartz-AlaLaurila-Labs/sa-labs-extension
Zen	Zeiss	RRID:SCR_013672
Igor Pro	Wavemetrics	https://www.wavemetrics.com/products/igorpro; RRID:SCR_000325
Adobe Illustrator	Adobe	https://www.adobe.com/products/illustrator.html; RRID: SCR_010279
Fiji	ImageJ	https://imagej.net/software/fiji/; RRID:SCR_002285
Knossos	Knossos	https://knossos.app/; RRID:SCR_003582
Paraview	Kitware	https://www.paraview.org/; RRID:SCR_002516

## Highlights

- EM reconstructions show that Müller glia envelop all layers of retinal vasculature
- Gaps in glial sheaths permit neuronal contact in intermediate vascular plexus
- Calcium signals propagate through Müller glia across layers and extend into sheaths
- Retinitis pigmentosa compromises Müller-vessel coupling in the deep vascular plexus
