# Supplementary material for: Layer-specific anatomical and physiological features of the retina’s neurovascular unit
Source: Curr Biol. Author manuscript; Available in PMC 2025 Feb 27. (PMC11867051; doi:10.1016/j.cub.2024.11.023)
Supplement: mmc1 [file NIHMS2041803-supplement-mmc1.pdf]

**Current Biology, Volume *35***

## Supplemental Information

# Layer-specific anatomical and physiological features of the retina's neurovascular unit

**William N. Grimes, David M. Berson, Adit Sabnis, Mrinalini Hoon, Raunak Sinha, Hua Tian, and Jeffrey S. Diamond**

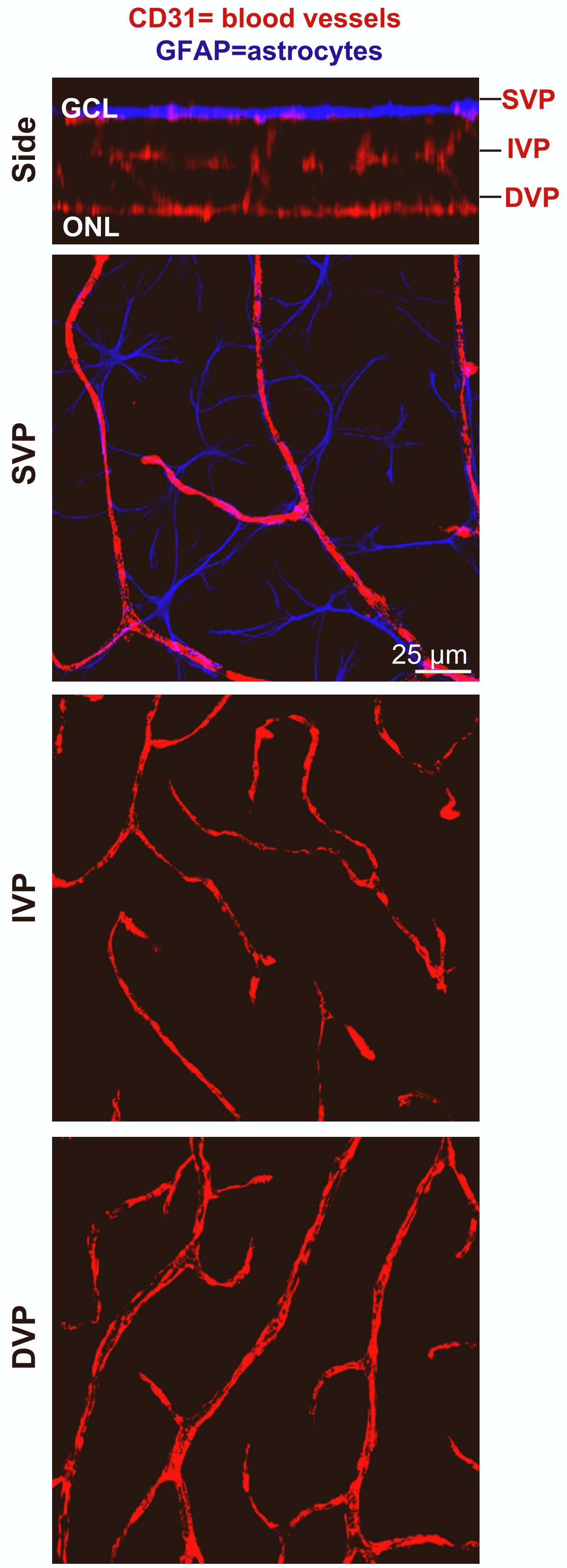


**Figure S1. Retinal astrocytes are confined to the superficial vascular layer. Related to Figure 1.** Immunohistochemistry labeling of retinal blood vessels (red, CD31) and astrocytes (blue, GFAP).

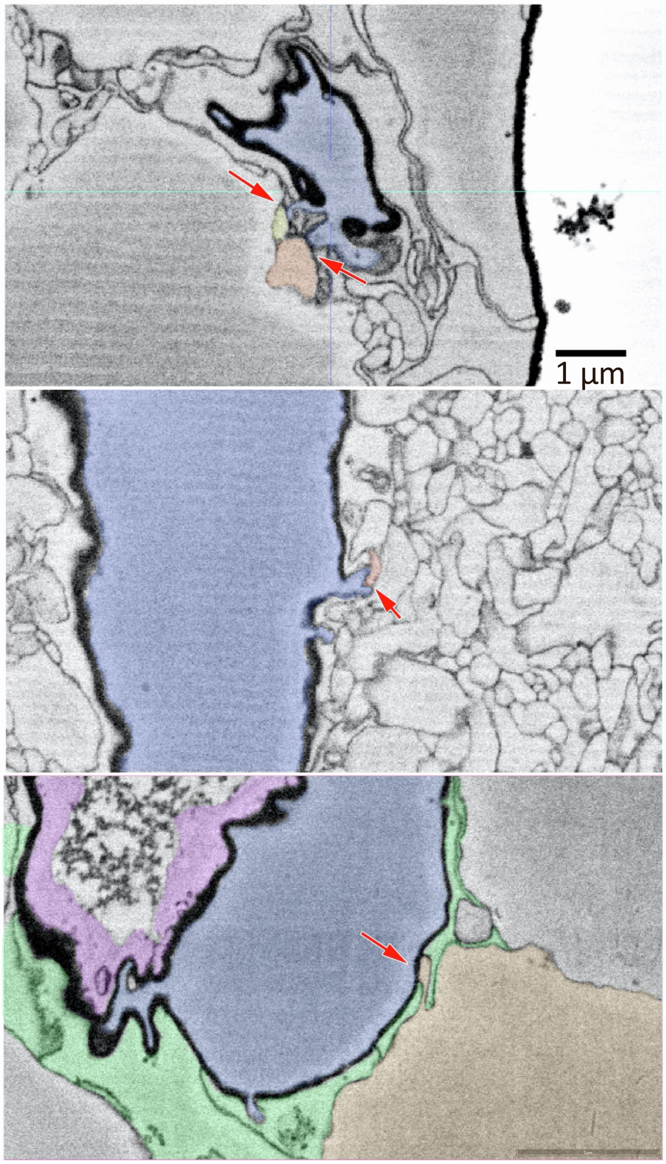


**Figure S2. Neuron-pericyte contacts in all three vascular layers. Related to Figure 4.**
(from the Helmstaedter volume)
*Top*, Contact of SVP pericyte protrusions (with two ganglion cell axons (yellow, orange) positively identified by tracing each axon back to a ganglion cell soma. Scale bar applies to all three panels. Volume coordinates: 7109, 3001, 2568.
*Middle*, Spine (red arrow) of an IVP pericyte soma (*blue*) contacting a fine neuronal process (*pink*). Note the thinning of the basement lamina around the spine. Volume coordinates: 4810, 5516, 1183.
*Bottom*, In the DVP, a protrusion from a bipolar cell soma contacts a pericyte soma (red arrow). In this panel, green tint marks Müller cell processes and pink marks endothelium. Volume coordinates: 2903, 3545, 609.

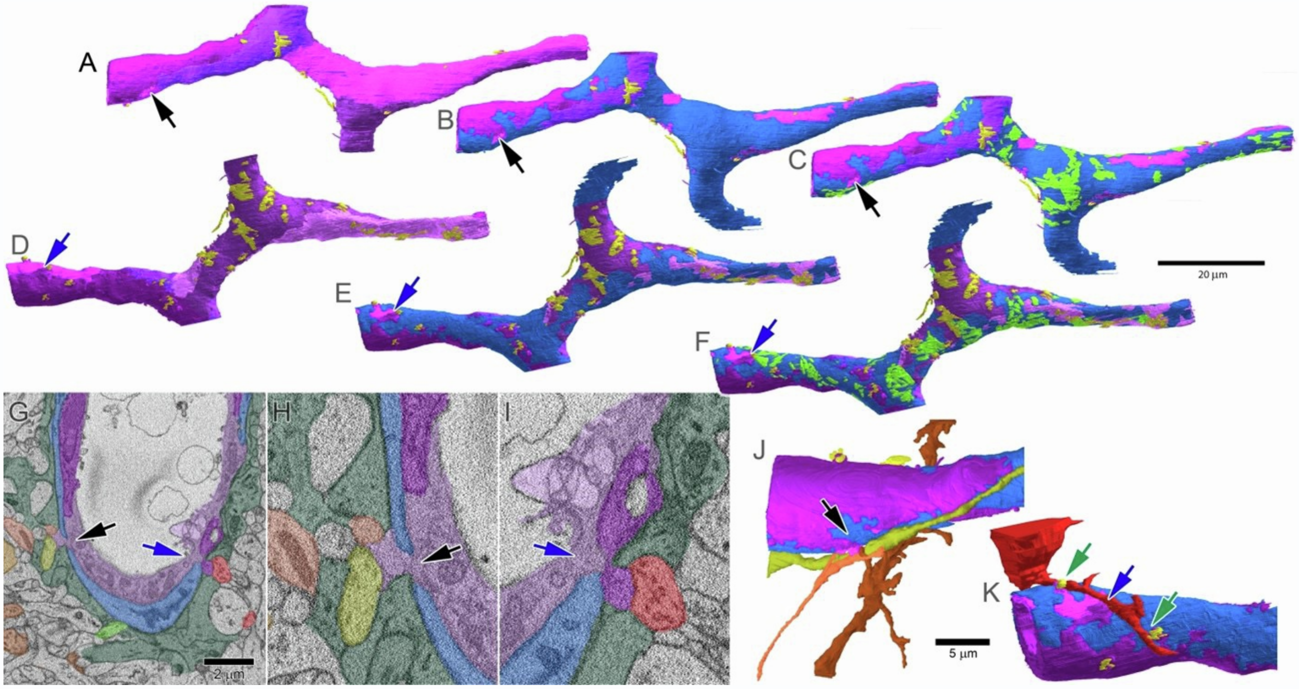


**Figure S3. Neuronal contacts onto a capillary in mouse somatosensory cortex. Related to Figure 4.**
(from the Gour volume)
Endothelial cells are *purple* and pericytes are *blue* in all panels.
(A-F) Six views of the capillary, highlighting different structural features. Top row (A-C) shows the vessel from one perspective and the bottom row (D-F) after rotation of the capillary around its long axis to reveal the reverse side. Left pair vessel reconstructions (A,D) show endothelial cells and their neuronal contacts (*yellow*). Individual endothelial cells are shown in slightly different shades to reveal intercellular junctions. Middle pair of views (B,E): same as A,D but with the addition of the pericyte (*blue*). Right views (C,F): same but with the addition of neuronal contacts to the pericyte (*green*). The largee patch of neuronal contacts in C marks the location of the pericyte's soma. An endothelial soma appears in D (center) marked by large patches of neuronal contact (yellow). Note that neuron-to-endothelial contacts are concentrated on one side of the capillary (D-F) and (except near the soma) cluster near distal pericyte processes. They are also concentrated at intercellular junctions between endothelial cells, especially on short endothelial spines, but also on long somatic spines (E; middle).
(G-I) Electron micrograph drawn from a single plane of the dataset passing through the same capillary (coordinates: 1680, 832, 538); with selected cellular profiles tinted to highlight features of interest. Endothelium: *purple*; pericyte: *blue*; astrocytes forming vascular sheath: *dark green*; neurons contacting endothelium: *warm colors*; neuron contacting pericyte: *green*. *Black arrow* in G and in the expanded view in H marks the site of a short endothelial spine that skirts the edge of the pericyte and penetrates through the otherwise continuous astrocytic sheath (*dark green*) into the parenchema, where it receives direct contacts from several neuronal processes. This location is also marked by black arrows in the expanded view in H as well in the 3D reconstructions of A-C and J. *Blue arrow* in C (as well as D-F and K) marks the site of a similar endothelial spine with neuronal contacts; this spine derives from the other endothelial cell forming the capillary wall here. Both endothelial spines lie near the boundary between the two endothelials cells (two shades of purple), as well as near distal pericyte processes that receive local neuronal contact (C and F; arrows).
(J,K) Local volumetric reconstructions of the neuronal processes making contact at the sites marked in other panels by the black arrow (J) and blue arrow (K). These include presumed axons (*orange* and *olive* in J) as well as spiny dendrites (*dark orange* in J; proximal dendrite in *red* in K, with partial somatic reconstruction) which makes two additional contacts onto the endothelium (*green arrows*), one of them again at an intercellular junction between endothelial cells (right green arrow).

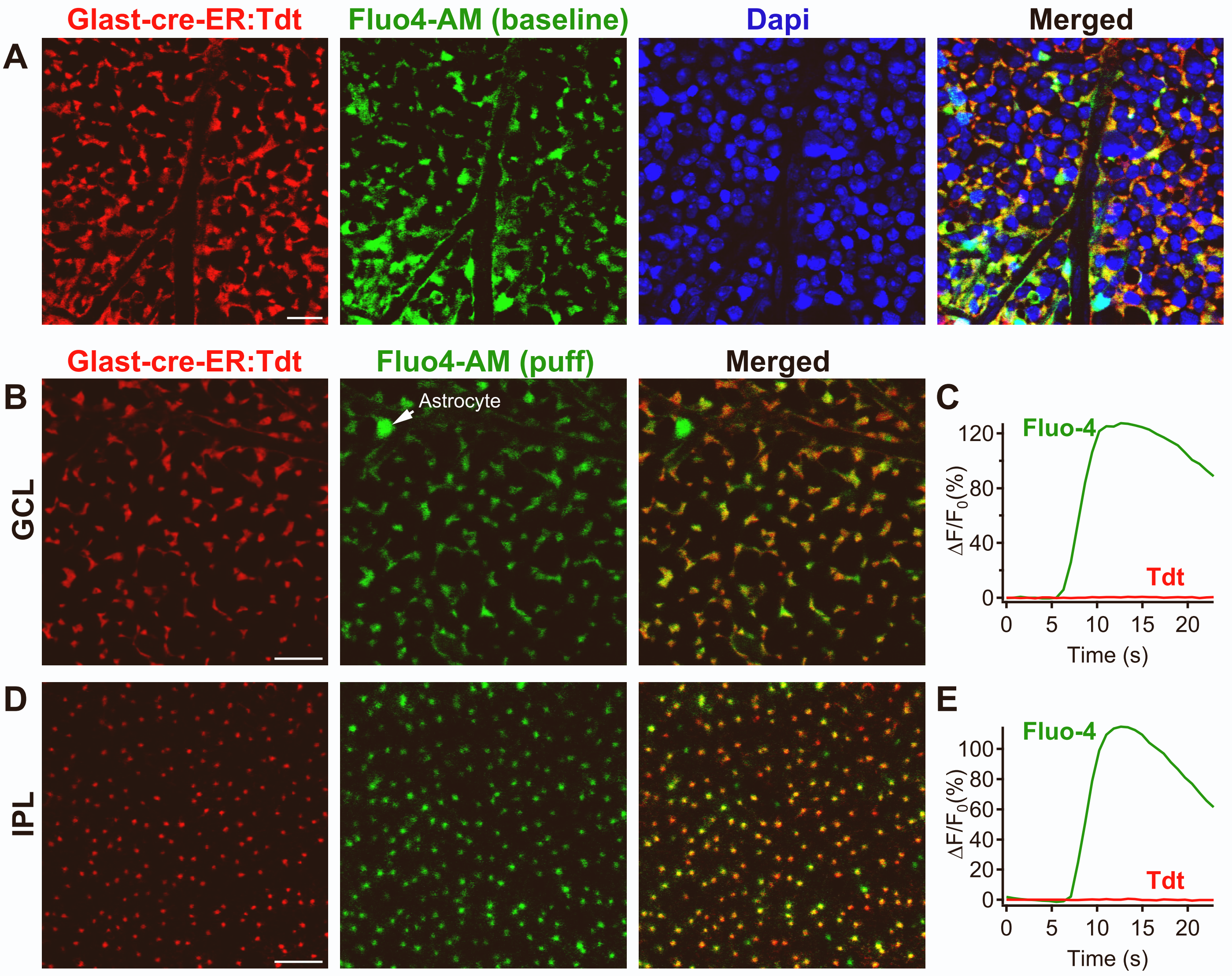


**Figure S4. Fluo4-AM loading protocol labels astrocytes and Müller glia but not neurons. Related to Figure 5.**

(A) Triple labeling of the SVP/GCL in the Glast-cre-ER:Tdt retina. Previous studies have shown this line selectively labels for Müller glia (Biesecker et al., 2016; Tworig & Feller, 2021; Wang et al., 2017). Tamoxifen-induced Tdtomato(Tdt) expression in red. Fluo4-AM loading in green. DAPI, a nuclear stain, is shown in blue. Far right image is a merge of the 3 labels.

(B) ATP puff-evoked signals in the GCL. Glast expression in red, baseline-subtracted puff response in green, and a merge of the two labels.

(C) Time course of the puff-evoked response in the two channels demonstrates a lack of channel bleed-through.(D,E) Same as B-C but within the IPL near the IVP. All scale bars are 20 μm.

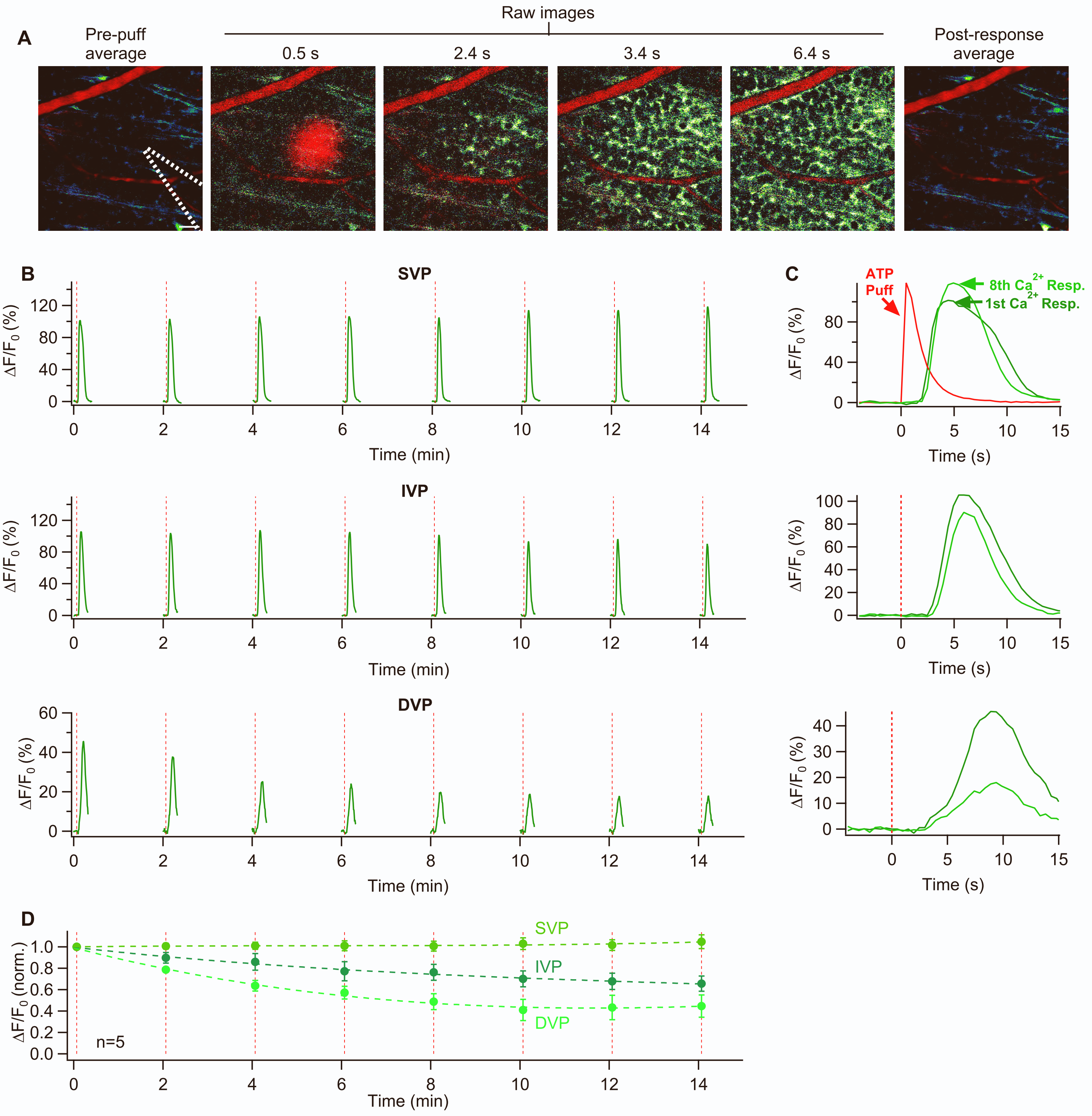


**Figure S5. Timing of Fluo-4 signals relative to ATP puffs. Related to Figure 5.**

(A) Sequence of images showing the relative time course and spread of the puff versus the Müller -derived Ca signal. First and last images are the average of 6 frames before the puff and after the Ca response, respectively. Middle images are raw images from single time points (time noted above each image). Vessels are labeled with SR-101, glia are labelled with Fluo-4 AM and the puff electrode (location indicated with dashed white lines) is filled with 5 mM ATP and Alexa 647. Scale bar is 20 µm.

(B) ΔF/F responses to 8 sequential puffs, delivered to the retinal surface every 2 minutes, in the (top) SVP, (middle) IVP and (bottom) DVP. The timing of the puff onsets is indicated by dashed red lines.

(C) Expanded view of the first (dark green) and eighth (light green) response in the sequence from the (top) SVP, (middle) IVP and (bottom) DVP. Top plot also shows the time course of the puff (red, i.e. Alexa 647). Red dashed lines in the middle and bottom plots indicates the onset of the puff stimulus.

(D) Pooled data showing the puff responses over time in each of the three layers. Responses were normalized to the initial response before averaging across samples (mean ±SEM, n=5).

| SBFEM Volume | Species | Tissue | Size (µm³) | Total Cap. Length Analyzed (µm) | Reconstruction Availability* |
|---|---|---|---|---|---|
| **Ding** | Mus Musculus | retina | 50 × 210 × 260 | 20 | WebKnossos |
| **Helmstaedter** | Mus Musculus | retina | 130 × 80 × 120 | | WebKnossos |
| **Palotto** | Mus Musculus | retina | 73 × 28 × 58 | 5 | Dryad |
| **Gour** | Mus Musculus | neocortex | 76 × 92 × 78 | 80 | WebKnossos |
| **Motta** | Mus Musculus | neocortex | 62 × 95 × 93 | 60 | WebKnossos |
| **Hoon** | Macaca Nemestrina | retina | 90 × 90 × 90 | 10 | Available upon request |

**Table S1. SBFEM volumes used in this study. Related to Figures 2-4.**
Provided links point specifically to analyzed regions of each data set.
